# Pelvic Organ Prolapse-Associated Cystitis

**DOI:** 10.1007/s11884-014-0249-4

**Published:** 2014-06-25

**Authors:** Rizwan Hamid, Giovanni Losco

**Affiliations:** 1 Department of Neurourology, Royal National Orthopaedic Hospital, Stanmore, UK; 2 Department of Urology, University College Hospital, 235 Euston Road, London, NW1 2BU UK

**Keywords:** Prolapse, Pelvic organ prolapse, Cystocele, Vagina, Urinary bladder, Rectocele, Urinary tract, Urinary tract infections, Bacteriuria, Pyuria, Cystitis, Urinary incontinence, Urinary incontinence, stress, Urinary incontinence, urge, Surgical mesh, Anti-bacterial agents, Antibiotic prophylaxis

## Abstract

Pelvic organ prolapse (POP) and urinary tract infection (UTI) are important problems, estimated to affect around 14 and 40 % of women, respectively, at some point in their lives. Positive urine culture in the presence of symptoms is the cornerstone of diagnosis of UTI and should be performed along with ultrasound assessment of postvoid residual (PVR) in all women presenting with POP and UTI. PVR over 30 mL is an independent risk factor for UTI, although no specific association with POP and UTI has been demonstrated. The use of prophylactic antibiotics remains controversial. The major risk factors for postoperative UTI are postoperative catheterisation, prolonged catheterisation, previous recurrent UTI and an increased urethro-anal distance—suggesting that global pelvic floor dysfunction may play a role.

## Introduction

Pelvic organ prolapse (POP) and urinary tract infection (UTI) are common problems. Urinary tract infection (UTI) will affect around 40 % of women in their lifetime, and a recurrent UTI will affect around 27 % within the following 6 to 12 months. The annual incidence of surgery for POP is up to 50 cases per 10,000 women-years. This is expected to double in the next 30 years [1].

A definition for recurrent UTIs is not universally accepted. Two, three, or four or more [2–4] UTIs in the preceding 12 months are some of the commonest definitions. There has been no comprehensive study of recurrent UTIs in large groups, across all ages, or for their relationships and associations to a wide range of urodynamic and clinical parameters. In particular, their relationship to POP is not well documented in the literature despite the common occurrence of these two entities in older females and the presence of a possible cause and effect mechanism in some cases.

In the previous literature, the separation of risk factors for recurrent UTI in women has tended to be on the basis of age. Commonly, three groups are identified: young premenopausal women, menopausal women between 50 and 70 years, and elderly women. Most studies to date reflect these patient groups [5].

There are a number of factors associated with young women and recurrent UTI. Most commonly these are sexual intercourse [6, 7], which includes a new sexual partner in the previous 12 months [6]; the use of contraceptives, especially spermicides, diaphragms and oral contraceptives [6, 8, 9]; family history [10]; and urethro-anal distance (UAD) [11]. The increased susceptibility of women with recurrent UTI to vaginal colonisation with certain uropathogens compared to women without recurrent UTI has been the focus of other studies [12]. The prevalence of recurrent UTI in women who are non-secretors of ABO blood group antigens has been found to be up to four times higher than in secretors (uropathogenic *Escherichia coli* have superior adherence to the uroepithelial cells of non-secreting women, hence an elevated risk of clinical infection in these women) [13].

Postmenopausal women have a different array of common risk factors for recurrent UTI. These are UTI history before menopause, oestrogen deficiency, surgery to the urogenital region, cystocele, elevated postvoid residual (PVR), and previous UTI [14, 15]. Cystocele, with its association with elevated PVR, means a number of women with POP will concomitantly present with recurrent UTIs. This presents a management dilemma. Does the presence of prolapse contribute to UTI? Will management of the prolapse decrease the risk of UTI? Is there an associated risk of postoperative UTI? In this review, we look at the current evidence regarding POP and associated cystitis or UTI.

## What Is POP?

POP affects a significant number of women in the UK, with anywhere from 11 to 14 % requiring surgical intervention at some point in their lifetimes [16, 17]. The USA alone performs approximately 200,000 pelvic prolapse surgical procedures per year [18]. Women may present with the sensation of dragging or a lump in their vagina, with sexual dysfunction, or with bladder and/or bowel dysfunction. In a small number of patients, recurrent UTIs may be the single presenting complaint.

Proper pelvic floor anatomy and prevention of POP are reliant upon maintaining the proper anatomical support mechanisms [19••]. The bony pelvis is composed of two hip bones connected posteriorly by the sacrum and anteriorly by the pubic symphysis. The female pelvis is wider than in the male so as to accommodate childbirth; however, this creates a predisposition for pelvic floor weakness. The pelvic diaphragm is composed of the levator ani and coccygeus muscles. It is the fusion of the posterior fibres of the pubococcygeus muscles with the iliococcygeus muscle that forms the levator plate, a structure between the anus and the coccyx that the pelvic organs rest upon. Levator ani weakness may lead to a sagging of the levator plate, which opens the urogenital hiatus and predisposes to POP [19••]. The endopelvic fascia is a dense collection of collagen, elastin, smooth muscle, fibroblasts and vessels that connects the bladder, urethra, vagina and uterus to the pelvic sidewall [20]. It is this endopelvic fascia, surrounding the vagina and connecting laterally to the arcus tendineus fascia, that provides pelvic sidewall attachment as well as support to the vaginal walls [21].

Three levels of connective tissue support have been described for the vagina. Level I support consists of the uterosacral ligament, connecting the cervix and upper vagina. This inserts onto the pelvic sidewall and sacrum. It is at this level that integrity of the vaginal apex is maintained, which is now understood to be critically important in repair. Level II support maintains anterior vaginal functional anatomy. It is composed of the pubocervical fascia along the length of the vagina. Finally, level III support maintains distal function and anatomy. This consists of the perineal membrane, perineal body and the superficial and deep transverse perineal muscles [19••, 21].

Defects at any of these levels, and usually a combination of two or all three of them, result in typical anterior compartment prolapse (cystocele), which may predispose to UTIs. Rarely, a significant posterior compartment prolapse (rectocele) may press anteriorly on the bladder neck and urethra causing obstructive voiding, urgency/frequency and/or elevated postvoid residuals, in turn raising the possibility of recurrent UTI.

The scope of POP repair is beyond this article, but the best practice methods are based on restoring normal vaginal anatomy to maintain adequate vaginal axis, length and function (see Table 1). Multiple surgical approaches have been described, including transvaginal repairs (with and without mesh), sacrospinous fixation, abdominal sacrocolpopexy, laparoscopic sacrocolpopexy and robotic sacrocolpopexy. Regardless of the location of defect, it is becoming evident that of critical importance for a durable and effective repair is the adequate fixation of the vaginal apex. The Surgery for Pelvic Organ Prolapse Committee has described the apex as the “keystone of pelvic organ support” and further emphasises that “the best surgical correction of the anterior and posterior walls may fail unless the apex is adequately supported” [21, 22, 23•].

**Table 1:** Types of prolapse repair by surgical approach

Surgical approach	Type of repair
Conservative	Ring pessary
Transvaginal approach	Anterior colporrhaphy
	Posterior colporrhaphy
	Mesh repair
	Sacrospinous fixation
Abdominal approach	Open sacrocolpopexy
	Laparoscopic sacrocolpopexy
	Robotic sacrocolpopexy

## What Is Cystitis?

Broadly, UTI or cystitis is defined as an infection of the urinary system and may involve the lower urinary tract or both the lower and upper urinary tracts [24]. It ranges on a spectrum from asymptomatic bacteriuria, to symptomatic UTI, to urinary sepsis requiring hospitalisation (see Table 2) [25, 29••]. Asymptomatic bacteriuria in women is defined as presence of at least 10^5^ CFU/mL of the same uropathogen in two consecutive clean-catch midstream urine samples obtained from patients without any symptoms or signs attributable to urinary infection. Asymptomatic bacteriuria is a state of colonisation, not disease, and does not indicate an infection that requires treatment [26–28].

**Table 2:** Types of UTI and their definitions

Condition	Definition
Asymptomatic bacteriuria	Presence of at least 10^5^ CFU/mL of the same uropathogen in two consecutive clean-catch midstream urine samples obtained from patients without any symptoms or signs attributable to urinary infection
Symptomatic UTI	Symptoms and signs of UTI and laboratory tests confirming the diagnosis (bacteriuria of at least 10^5^ CFU/mL and pyuria of at least 10 white blood cells per high-powered field)
Uncomplicated UTI	UTI in a normal urinary tract without prior surgery or instrumentation
Complicated UTI	UTI where there is a functional or structural abnormality, previous urinary instrumentation or surgery, systemic diseases such as renal insufficiency, diabetes or immunodeficiency, or previous renal transplant
Urosepsis	Sepsis due to UTI

Establishing a diagnosis of symptomatic UTI requires a patient to have symptoms and signs of UTI and laboratory tests confirming the diagnosis (bacteriuria of at least 10^5^ CFU/mL and pyuria of at least 10 white blood cells per high-powered field). Uncomplicated symptomatic UTI is present when there is a symptomatic bladder infection manifested by dysuria, worsened urinary frequency or urgency, fever, suprapubic tenderness, costovertebral angle tenderness with no other cause attributable and laboratory tests revealing UTI [29••]. Fever is usually not present in symptomatic UTI that is localised to the bladder. Complicated UTI is defined as having a symptomatic UTI in patients caused by a functional or structural abnormality, having had urinary instrumentation, having systemic diseases such as renal insufficiency, diabetes, or immunodeficiency or having undergone renal transplantation [30–33]. Pyuria is simply defined as the presence of leukocytes in the urine.

## Investigations for UTI

Most patients, but not all, will present with dysuria, frequency, urgency, nocturia and urge incontinence. Malodorous urine and haematuria may be present. Very elderly patients, and patients who are neurologically impaired, may present differently, and a high level of suspicion should be had for UTI. Patients who are reliant on self-catheterisation in order to void may suffer only from lower abdominal pain or systemic malaise in the absence of specific lower urinary tract symptoms. Fever is uncommon in the first instance but in some patients will be the only symptom. However, when superimposed with a high prevalence of chronic genitourinary symptoms, increasing cognitive impairment and a high comorbidity load with advancing age, diagnosis and management of a symptomatic UTI remains a challenge [29••].

Clinical examination, including the formal examination for prolapse, should be performed.

The cornerstone of UTI diagnosis is the presence of a positive urinary culture. For this reason, a urine specimen should always be sent for microscopy, culture and sensitivity. Irritative bladder symptoms, not due to infection, must be ruled out by the presence of a positive urine culture before the patient is considered to have a UTI. Documented positive cultures become important when counselling patients as to whether treatment of their prolapse will have any effect on their symptoms. Imaging should include ultrasound assessment of the bladder and kidneys, including an assessment of postvoid residual.

## UTI in Association With POP

Haylen et al. conducted an interesting prospective study of 1,140 women presenting for routine urogynaecological assessment due to symptoms of pelvic floor dysfunction. All patients underwent urodynamic studies [34]. Significant risks for an increased prevalence of recurrent UTI in women were found to be nulliparity and a PVR over 30 mL.

This appears to be the first time that nulliparity and recurrent UTI have been so clearly linked. A mechanism was proposed by the authors whereby parity could in fact protect against recurrent UTIs. As parity increases, number of sexual partners may decrease. Furthermore, new mothers may be less likely to engage in intercourse due to the stresses that a new child brings [5–9]. The effect of pregnancy and childbirth on relaxing and stretching the birth canal may help in preventing UTIs: if the friction of intercourse has a role in promoting recurrent UTIs, this would have a lesser impact postpartum [34]. It is known that sexual histories are difficult to study in a research context due to the difficulty in obtaining them making this theory difficult to prove.

An elevated PVR is another significant factor associated with recurrent UTIs (over 30 mL). The level of PVR, described by O’Grady and Cattell, at which UTI becomes more frequent, was 30 mL [35]. An upper limit of normal PVR of 30 mL is supported if measured immediately after voiding by an accurate ultrasound method [36, 37]. An ultrasound PVR assessment is therefore recommended for women with recurrent UTI.

Voiding dysfunction is commonly seen with anterior compartment prolapse and is often under-recognised. In the Haylen paper, women with a final diagnosis of voiding dysfunction had an increased chance of recurrent UTI. This diagnosis included an assessment of PVR, flow rate and pressure-flow studies [34]. Up to 39 % of women attending for assessment of the urogenital tract have been found to have a preliminary diagnosis of voiding dysfunction, where slow or incomplete micturition is included as diagnostic criteria [38]. The final diagnosis and the quest for a cause rely upon formal urodynamic assessment.

Interestingly, in the Haylen paper, no specific association with UTI and prolapse was seen. An increasing negative association between recurrent UTI and prolapse of higher grade is difficult to explain. The link between voiding dysfunction and prolapse has been documented [38]. Despite the link between voiding dysfunction and recurrent UTI, there is an inverse relationship between prolapse and recurrent UTI. It is possible that due to the increased risk of recurrent UTI in nulliparous women where the stretching effects of childbirth are not found that this further increases the apparent risk—whereas in parous women with prolapse, the stretching effects have obviously been marked, decreasing the UTI risk as detailed earlier [39].

## Postoperative UTI

After surgery for pelvic organ prolapse (POP) or stress urinary incontinence (SUI), UTIs are the most common complication [40, 41]. By the age of 80 years, up to one in nine women will seek surgery for POP and SUI due to their prevalence in women [42, 43]. A major trial where autologous sling was compared to Burch colposuspension for treatment of SUI was known as the SISTEr trial. In the sling group, 48 % of women suffered a UTI compared to 32 % in the Burch group during the first 24 months of follow-up [40]. In a further study in the Medicare population, where 1,356 sling procedures were analysed, a UTI developed in 33.6 % of women within 3 months of surgery [44]. When considering obliterative surgery for advanced prolapse in older women, 45 % of women developed a UTI within 3 months of surgery [41]. This is a very high occurrence of a significant adverse event for many women; however, given the instrumentation of the urinary tract from catheters and cystoscopy inherent in many prolapse operations, it is understandable. In order to reduce postoperative UTI, targeted strategies are needed. Risk factors that can be modified need to be identified.

In a case-control study by Sutkin et al., the risk of UTI within 6 weeks of surgery for POP or SUI was 9.0 % [45•]. This compares to the SISTEr trial, mentioned above, where almost 17 % of women were treated for UTI within 6 weeks of surgery [46]. Two factors, which were found to increase the risk of UTI postsurgery, were transurethral catheterisation and incomplete bladder emptying [47]. These findings were in spite of the use of prophylactic antibiotics.

One of the bladder’s natural mechanical defences against UTIs is its ability to empty; however, this is compromised by the transient urinary retention that can occur commonly after pelvic floor surgery [48, 49]. There are a number of causes for postoperative retention including anaesthesia, swelling and oedema of tissues, denervation, devascularisation and opioid use. The subsequent placement of a catheter to empty the bladder, which is frequently left in situ, is well known to increase the risk of UTI [50]. In the SISTEr trial, catheterisation after a failed trial of void resulted in a more than two-fold increase in risk of UTI [46]. Another study found a more than four-fold increase in risk for postoperative UTI in women who failed a trial of void and subsequently had a catheter placed for more than 10 days [47].

When defining an acceptable trial of void, a commonly used range of PVR in clinical practice is 75–150 mL [45•]. For women with preoperative PVRs greater than 200 mL (and in some cases much higher), women with severe prolapse, and for those being discharged on the day of surgery, such a range may be inappropriate. With a trend towards day of surgery discharge, the shorter postoperative period may not provide sufficient time for the resolution of urinary retention. Indeed, there may be a rush for a clinical decision to be made about the need for catheterisation with a tendency to place a catheter if there is any doubt, in order to reduce the patient having to re-present to hospital after hours. This situation therefore presents a clinical challenge. In order to reduce postoperative UTI, a balance needs to be found between appropriate but not overzealous use of catheters. This should be tailored to the individual patient taking into account her age, mobility, type or surgical repair, presence of preoperative recurrent UTIs and preoperative PVRs [51••].

Paradoxically, Sutkin et al. reported that the risk of UTI was increased by a larger UAD (adjusted OR 1.4). The POP-Q examination, where the genital hiatus and the perineal body are measured, allowed the hypothesis that a decreased UAD would increase enteric colonisation of the bladder to be tested. At first thought it appears counterintuitive that increasing the distance between the exit and entry points of enteric organisms would increase the risk of UTI. However, the authors proposed that this increased UAD is a surrogate marker for global pelvic floor neurological and muscular compromise, which manifests itself as prolapse—descent of the perineum and pelvis [52]. As the neuromuscular compromise and pelvic floor dysfunction is unchanged after prolapse surgery, despite the fact that the UAD will decrease, the risk of urinary stasis and UTI development may remain in these women.

There are no studies that we could find in writing this review that tested the effect of prolapse treatment on reducing the risk of subsequent UTI. Whilst reducing and repairing prolapse may assist in reducing PVR, a well-established factor in the development of UTIs in these patients, the role of global pelvic floor dysfunction should not be discounted. Therefore, it is likely that surgical treatment of prolapse alone will be insufficient to prevent UTIs for many women. More research in this area is warranted.

Postoperative use of prophylactic antibiotics is controversial. Routine antibiotics to prevent UTI in women with asymptomatic bacteriuria associated with catheter use are not generally recommended [53–56, 57••]. In a randomised trial of women with suprapubic catheters placed for incomplete emptying after prolapse surgery, nitrofurantoin prophylaxis was shown to decrease the rate of symptomatic UTIs [58].

Further research to explore urinary acidifiers and other non-antibiotic measures such as vaginal oestrogens and other nutriceuticals are required.

## Conclusions

POP and UTI are both common problems, causing considerable morbidity for women. Whilst both problems occur frequently in isolation, there is a considerable cohort of women who suffer from both. Clinical examination and an ultrasound assessment of PVR should be performed in all women presenting with prolapse and UTI. Elevated PVR is the most significant risk factor, linking POP with UTI. The major risk factors for postoperative UTI are postoperative catheterisation and prolonged catheterisation, as well as previous history of recurrent UTI and increased UAD. There is a need for more evidence on the use of catheters to manage transient postoperative retention, as well as more research to find ways of preventing UTI in this group.
